# Acceptability of Telehealth Physical Therapy in Adults with Chronic Low Back Pain: A Multivariable Analysis of Knowledge, Attitudes, Barriers, and Biopsychosocial Health Factors

**DOI:** 10.3390/healthcare14142036

**Published:** 2026-07-08

**Authors:** Yousef M. Alshehre

**Affiliations:** Department of Health Rehabilitation Sciences, Faculty of Applied Medical Sciences, University of Tabuk, Tabuk 47512, Saudi Arabia; yalshehre@ut.edu.sa

**Keywords:** telehealth, physical therapy, low back pain, acceptability, knowledge, attitude, barriers

## Abstract

**Background:** Telehealth acceptance for chronic low back pain (CLBP) remains unclear. It may depend on patients’ knowledge, attitudes, barriers, and health factors. This study assessed telehealth physical therapy’s acceptability for CLBP by examining willingness, knowledge, attitudes, and barriers, and comparing willingness among healthcare professionals. **Methods:** This study included 309 adults with CLBP. The Telemedicine Perception Questionnaire (TMPQ) assesses perceptions and barriers. Participants rated telehealth willingness and completed the PROMIS-29 for health factors. The descriptive statistics summarized the characteristics. The Friedman test was used to compare professional willingness, *t*-tests were used to examine sex differences, Pearson’s correlation was used to assess PROMIS-29 and telehealth willingness, and multivariate regression was used to identify the related variables. **Results:** The participants had an average age of 26.06 years, and 90% were female. Willingness varied (*p* < 0.001), being highest among dietitians (3.86 ± 1.15), lowest in physical therapists (3.52 ± 1.13). Males reported more telehealth barriers (*p* = 0.043), and females had higher anxiety, depression, fatigue, and pain scores (*p* < 0.05). Willingness to use telehealth physical therapy correlated with physical function (r = 0.319). The knowledge score was most strongly correlated with physical function (r = 0.368). Regression analysis showed that knowledge (B = 0.057, *p* = 0.011) and attitude (B = 0.063, *p* < 0.001) predicted greater telehealth willingness, with the model explaining moderate proportion of the variance in willingness to use telehealth physical therapy (R^2^ = 0.381; adjusted R^2^ = 0.370). **Conclusions:** Telehealth physical therapy was moderately acceptable in adults with CLBP. Knowledge and acceptance increased the willingness to use telehealth services. Higher knowledge of telehealth and more positive attitudes were associated with a greater willingness to use telehealth physical therapy. These findings suggest that patient education and clear orientation to telehealth procedures may support readiness for telehealth-based physical therapy. However, the strong predominance of female participants (90%) and the self-selected online sample should be considered when interpreting the findings, as these factors may limit generalizability to the broader CLBP population.

## 1. Introduction

Low back pain is a common musculoskeletal disorder and continues to be the leading cause of reduced activity and quality of life (QoL) worldwide [1]. Existing research is emphasizing non-pharmacological intervention for low back pain. This is due to limited long-term benefits and the known risks of opioid treatment [2,3,4]. Within this framework, physical therapy plays a key role, providing exercise-based, educational and functional interventions that can reduce pain and disability [5,6].

Although physical therapy is widely recommended, access to this is often hampered by transportation, costs, and geographical isolation [7,8]. These challenges have focused interest in telehealth as an alternative method to address these difficulties. Telehealth refers to the remote provision of physical therapy services using communication technologies such as videoconferencing, mobile applications, web-based systems and related digital tools [9,10]. The rapid expansion of telehealth during and after the COVID-19 pandemic has accelerated its acceptability and interest in its role in the care of low back pain [11].

Recent evidence in musculoskeletal rehabilitation indicates that telehealth physical therapy can be feasible, safe, and clinically acceptable for selected musculoskeletal conditions, particularly when supported by structured education, remote monitoring, appropriate digital infrastructure, and hybrid care models when in-person assessment is required [12].

However, physical therapy requires specific consideration because it differs from general telehealth consultations. Unlike consultation-based services, telehealth physical therapy often involves functional examination, exercise prescription, functional task monitoring, exercise correction, safety guidance, and patient confidence in completing therapeutic activities without direct physical contact with the therapist. Therefore, willingness to use telehealth physical therapy may be influenced by different concerns than willingness to use telehealth for general medical advice or counseling [13].

However, effectiveness alone does not guarantee patient acceptance. Physical therapy is often associated with direct observation, home instruction and supervised intervention by physical therapists. The success of telehealth physical therapy, therefore, depends not only on the availability of remote services, but also on the patient’s willingness to take part in this care. Acceptability may be influenced by perceived usefulness, knowledge of telehealth, trust in technology, privacy concerns, and wider attitudes towards remote consultation [14]. Telehealth acceptability can also be understood using established theoretical frameworks. The Theoretical Framework of Acceptability explains acceptance in terms of affective attitude, perceived burden, perceived effectiveness, self-efficacy, and understanding of the intervention. Similarly, the Technology Acceptance Model and Unified Theory of Acceptance and Use of Technology emphasize perceived usefulness, ease of use, facilitating conditions, and user confidence as key factors influencing technology adoption. These frameworks are relevant to telehealth physical therapy because patients must understand the purpose of remote care, perceive it as useful and safe, and feel confident in performing therapeutic exercises through digital platforms [15]. In this study, biopsychosocial health factors were measured using the PROMIS-29 health profile questionnaire, which captures physical function, pain interference, fatigue, sleep disturbance, anxiety, depression, social participation, and pain intensity in a standardized way. Including these domains was clinically relevant because such factors may influence patients’ confidence, perceived need for therapist support, and their readiness to participate in telehealth-based physical therapy.

These issues are particularly relevant for adults with CLBP. Although this group may benefit from remote access to supervised physical therapy and follow-up, it remains unclear whether telehealth physical therapy can truly serve as an alternative to in-person care. Its acceptability may also depend on patients’ perceptions, perceived barriers, and physical function. A better understanding of these links is essential to developing patient-oriented digital physical therapy pathway. However, most telehealth acceptability studies in musculoskeletal rehabilitation have examined mixed patient groups, have not focused specifically on adults with CLBP, and rarely integrate patients’ knowledge, attitudes, perceived barriers, and biopsychosocial health factors within a single analytical model [11,12,14]. Consequently, the specific unanswered question remains as to which of these factors are independently associated with willingness to use telehealth physical therapy among adults with CLBP, and whether willingness differs according to the type of healthcare professional providing telehealth care [8]. Therefore, the study aimed to assess the willingness to use telehealth physical therapy as a distinct rehabilitation service among adults with CLBP, to assess the knowledge, attitudes, and perceived barriers associated with telehealth, to compare the willingness of different types of healthcare professionals and to identify factors related to the willingness to use telehealth physical therapy. Based on the proposed rationale, I hypothesized that the willingness to use telehealth would differ across healthcare provider categories, with lower willingness for telehealth physical therapy than for more consultation-based services. I further hypothesized that higher telehealth knowledge and more positive attitudes would be associated with greater willingness to use telehealth physical therapy, whereas higher perceived barriers and poorer biopsychosocial health status would be associated with lower willingness.

## 2. Materials and Methods

### 2.1. Study Design and Reporting Framework

This study utilized a cross-sectional, web-based survey design and reporting that followed the Strengthening the Reporting of Observational Studies in Epidemiology (STROBE) guidelines for cross-sectional studies and the Checklist for Reporting Results of Internet E-Surveys (CHERRIES), thereby improving transparency in relation to online recruitment, consent procedures, questionnaire administration, and response handling [16,17,18].

### 2.2. Study Setting and Recruitment

Data were collected using an online survey platform over a twelve-month period. Participants were recruited through an open and non-preferential approach using social media and mobile messaging applications, in particular X and WhatsApp, complemented by electronic mail, links to invitations and word of mouth. Before entering the questionnaire, potential participants were shown a landing page with information about the purpose of the study, eligibility criteria, the expected time to complete the questionnaire (10 to 15 min), and the contact details of the investigator. Electronic informed consent was obtained from the participants before they answered the online questionnaire. An electronic consent form was displayed on the first page of the online questionnaire. This approach was adopted because the study aimed to capture the telehealth rehabilitation experience of adults with CLBP, which is available in digital environments, and no complete sample of eligible participants was available.

### 2.3. Sampling Strategy and Justification

A convenient self-selection sampling strategy was applied. Because participation was voluntary and conducted online, the recruitment strategy may have introduced selection bias. Individuals who were more digitally literate, more familiar with online health services, more motivated to participate in health research, or more interested in telehealth may have been overrepresented. Therefore, the sample may not fully reflect the broader population of adults with CLBP in the general population. This approach was considered appropriate because the study was exploratory and focused on attitudes, willingness and perceived barriers to telehealth physical therapy rather than prevalence estimates from a population defined by a registry. In online health research, convenience sampling is often used when investigators want to effectively reach geographically dispersed respondents effectively, especially when the target population is defined by a current health complaint and the research question is related to digital participation [19,20]. However, as the selection was based on voluntary online participation, the resulting sample may have included more individuals who were younger, more digitally engaged and more receptive to telehealth, which would reduce representativeness and increase the potential for selection bias.

### 2.4. Sample Size Calculation

The sample size was estimated a priori using the Krejcie and Morgan formula for finite populations, which resulted in a target sample of 384 participants, based on the hypotheses set out in the study design. The final analysis sample consisted of 309 complete responses. Therefore, the sample obtained did not meet the initial prevalence target and may have reduced the accuracy of the descriptive estimates. However, the final sample was sufficient for the predicted multivariate linear regression model with five predictors, which exceeded the Green’s minimum for the overall multiple correlation test (N ≥ 50 + 8 m; minimum = 90 for m = 5) [21,22]. In addition, based on the achieved sample size (N = 309), five predictors, α = 0.05, and the observed model fit (R^2^ = 0.381; f^2^ = 0.616), the achieved power for detecting the overall regression model was >99%. Nevertheless, the lower-than-planned sample size may have reduced the precision of prevalence-style descriptive estimates and the ability to detect small associations or subgroup differences. Therefore, the findings should be interpreted primarily as analytical associations within the study population and not as exact population estimates.

### 2.5. Eligibility Criteria

Participants were eligible if they were 18 years or older, had a clinical diagnosis of low back pain from a healthcare professional, and reported current CLBP at the time of survey completion which was defined in the survey as “a back pain issue that has persisted at least three months and has resulted in pain on at least half the days in the past six months” [23], could understand and respond to the survey in the Arabic language, and provided electronic informed consent. Responses with incomplete data were excluded.

### 2.6. Survey Content and Outcome Measures

The questionnaire included sections on socio-demographic characteristics, history of low back pain, telehealth perception, willingness to use telehealth among health care professionals and biopsychosocial health factors.

Sociodemographic characteristics included demographic data, marital status, smoking status, educational attainment, and self-reported history of chronic illnesses or musculoskeletal disorders. Low back pain included current pain intensity, lowest and highest pain in the previous 24 h, time since the first episode of low back pain, duration of the pain, and frequency of symptoms in the previous 6 months. The intensity of pain was recorded using a numerical pain rating scale (NPRS). NPRS has demonstrated good-to-excellent test–retest reliability (ICC/SER-derived MDC ≈ 2 points) and strong construct validity against the VAS/VRS in CLBP, with responsiveness and a commonly reported minimal clinically important change of ~2 points (≈30%) [24]. Telehealth perception was assessed using the Telemedicine Perception Questionnaire (TMPQ) which was scored on a five-point scale from 1 (strongly disagree) to 5 (strongly agree). Based on the data set, the questionnaire yielded scores for knowledge (4 points), attitudes (11 points), and barriers (5 points) [25,26]. TMPQ validation studies and translations have reported acceptable psychometric properties (internal consistency Cronbach’s α ≈ 0.76; high test–retest ICC) [27]. Participants also rated their willingness to use telehealth in five groups of healthcare professionals: physical therapy, mental health, emergency treatment, family medicine, and dieticians. To facilitate comparison across areas, the scores for perception were normalized to a scale of 0–100. The Patient-Reported Outcomes Measurement Information System (PROMIS-29) was used to evaluate the outcomes, with higher scores signifying improved function. For PROMIS-29, *p*-values were calculated using the raw scores, b = T-scores (population mean of 50, standard deviation of 10) accessible at the Assessment Center^SM^ website: http://assessmentcenter.net. The impact score was determined as the sum of the reversed raw physical function, raw pain interference, and pain intensity scores [28]. The PROMIS-29 have demonstrated good test–retest reliability (ICC typically >0.7), excellent internal consistency (Cronbach’s α generally ≥0.8–0.9), and acceptable structural and construct validity across clinical populations with chronic conditions [29]. The survey was conducted in Arabic only. The Arabic wording of the TMPQ and PROMIS-29 items was reviewed by bilingual experts to ensure conceptual equivalence, cultural appropriateness, and clarity for Arabic-speaking participants. Where established Arabic wording was available, it was retained; otherwise, items were translated and reviewed to preserve the meaning of the original constructs, following the recommended principles for translation and cultural adaptation of patient-reported outcome measures [30]. Because the present study was not designed as a formal psychometric validation study, no separate validation of the Arabic versions was performed in the CLBP sample.

### 2.7. Procedures

Participants opened the survey link and first viewed an electronic information page describing the study aims, procedures, duration, nature of participation, and confidentiality protections. Only participants who provided electronic consent were allowed to complete the questionnaire. Completed responses were screened for eligibility criteria. Personal identifiers were removed before the analysis, and only the researcher had access to the dataset.

### 2.8. Ethical Considerations

This study was approved by the Local Research Ethics Committee, University of Tabuk, Tabuk, Saudi Arabia (approval No. UT-241-86-2023). This study was conducted in accordance with the ethical principles of the Declaration of Helsinki. Participation was voluntary, clear information was provided before enrolment, privacy and confidentiality were protected, and electronic informed consent was obtained before the survey was completed. Participants were also informed that they could decline participation or discontinue the study before submission without any adverse consequences.

### 2.9. Statistical Analysis

All analyses were performed using SPSS version 27.0. Descriptive statistics were used to summarize the characteristics of the participants, with categorical variables presented as frequencies and percentages and continuous variables as mean ± standard deviation. The TMPQ was scored on a five-point Likert scale and the composite domain scores for knowledge, attitudes and barriers were calculated. As these domain scores represented the sum of the individual measures, they were analyzed as approximately continuous variables. The willingness ratings of the five categories of providers were compared using the Friedman test. When the Friedman test was significant, post hoc pairwise comparisons were performed using Wilcoxon signed-rank tests with Bonferroni correction to identify differences between the provider categories. Sex differences in domain scores were investigated using independent *t*-tests on samples. The association between PROMIS-29 scores and willingness to undergo telehealth physical therapy was assessed using Pearson’s correlation. The predictors entered in the multivariable regression model were selected a priori based on the study objectives and conceptual relevance. Knowledge, attitude, and barrier scores were included as modifiable telehealth-related perceptual factors, while age and sex were included as basic demographic covariates. A multivariable linear regression model used to investigate variables independently related to telehealth willingness to undergo physical therapy. Assumptions for parametric analyses were checked, including normality, homogeneity of variance, linearity, homoscedasticity, multicollinearity, and influential observations for the regression model. For the linear model, the total fit was summarized by using the R^2^ function and adjusting the R^2^. Statistical significance was set at *p* < 0.05.

## 3. Results

### 3.1. Participant Characteristics

The demographics and clinical characteristics of the sample are shown in Table 1. The mean age of the participants was 26.06 ± 9.29 years. Most respondents were female (90.0), single (76.0), non-smoking (95.0) and university-educated at the least secondary level (64.0). The mean body mass index (BMI) was 24.12 ± 5.82 kg/m^2^. Although 17.2% of the participants reported a chronic illness and 42.4% reported a history of other musculoskeletal disorders, all reported having CLBP. The mean current pain intensity was 2.78 ± 2.03 and the mean worst pain intensity over the preceding 24 h was 2.78 ± 2.85. More than half of the respondents (58.9%) reported having experienced their first episode of low back pain in the previous 12 months. Regarding the frequency of symptoms in the previous six months, 49.4% reported painless than half the time, 23.7% at least half the time and 20.5% daily.

### 3.2. Telehealth Perceptions

The responses to the Telemedicine Perception Questionnaire (TMPQ) are presented in Supplementary Table S1. Within the knowledge domain, 62.4% of participants agreed or strongly agreed that telehealth could be used for diagnosis, and 51.2% agreed or strongly agreed that it could be used for assessment. Attitude-related items showed general perceptions: 84.5% agreed that telehealth could save time, 75.7% believed it could save money, and 69.9% considered it a suitable form of healthcare delivery. In contrast, the barrier domain revealed mixed results. Most respondents disagreed that telehealth would violate privacy (72.2%), yet a considerable proportion remained concerned about the lack of physical contact and trustworthiness of equipment.

### 3.3. Willingness Across Provider Types

As shown in Table 2, the willingness to use telehealth varied significantly across provider categories (Friedman test, *p* < 0.001). Post hoc pairwise comparisons with Bonferroni correction were conducted to determine the provider categories that differed significantly. The highest willingness scores were observed for dietitian services (3.86 ± 1.15) and family doctor consultations (3.84 ± 1.11), followed by mental health therapist services (3.66 ± 1.17). Lower scores were recorded for urgent care (3.54 ± 1.16) and physical therapy (3.52 ± 1.13) than for other services. These findings indicate that telehealth was generally acceptable across provider groups, although physical therapy was viewed less favorably than the other remote healthcare services considered in this study.

### 3.4. Sex-Based Comparisons

Sex-based comparisons are presented in Supplementary Table S2. Female participants had a slightly but statistically significantly higher knowledge score than males (14.3 ± 3.6 vs. 13.2 ± 3.3; *p* = 0.049). The attitude scores were comparable between females and males (42.0 ± 8.0 vs. 41.0 ± 7.0, *p* < 0.700). Male participants had a significantly higher barrier score than females (16.1 ± 2.5 vs. 14.7 ± 3.6; *p* < 0.043). There was no significant difference between the sexes in the willingness to use physical therapy in the telehealth setting (3.5 ± 1.1 vs. 3.4 ± 1.1; *p* = 0.452).

**Figure 1:**
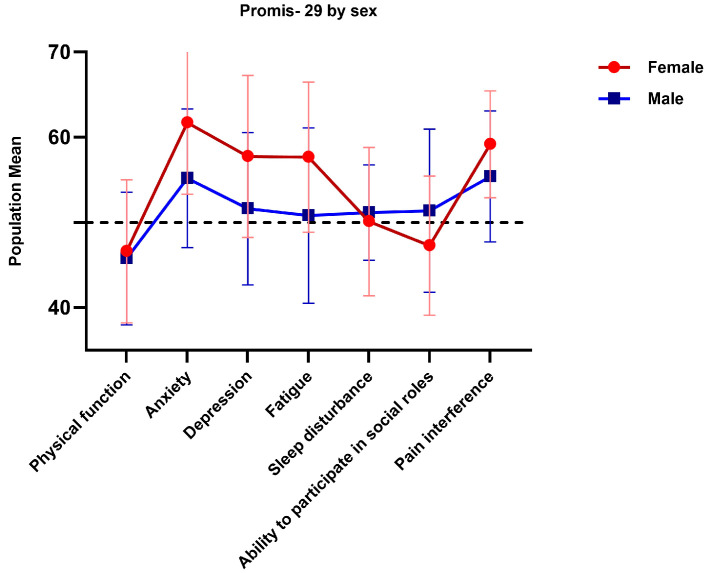
Mean PROMIS-29 between female and male.

PROMIS-29 v2.0 T-scores showed significant differences between sexes in several domains (Figure 1 and Supplementary Table S3). Females reported significantly higher anxiety, depression, fatigue, and pain interference scores than males (all *p* < 0.05), whereas males demonstrated a significantly better ability to participate in social roles (*p* = 0.026). No significant sex differences were observed in physical function, sleep disturbance, or pain intensity (*p* > 0.05).

### 3.5. Correlational and Multivariable Regression Findings

**Figure 2:**
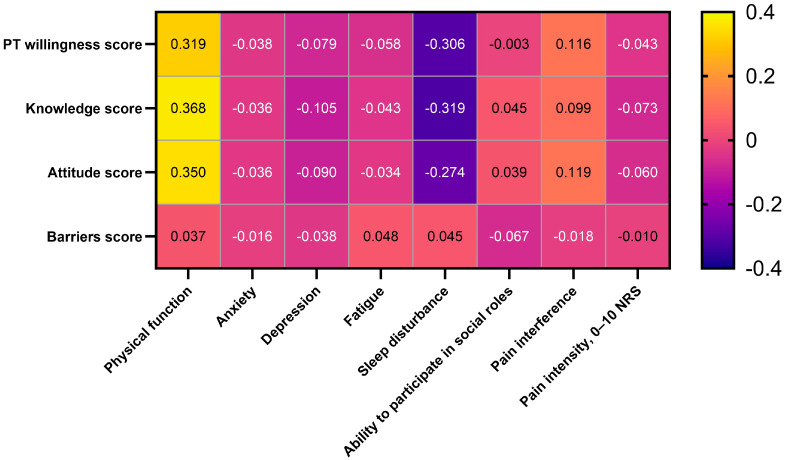
Heat map showing correlations between telehealth-related scores and PROMIS-29 domains. The color scale indicates the direction and strength of correlations: yellow represents positive correlations, dark purple represents negative correlations, and lighter/central tones indicate weak or near-zero correlations. For grayscale interpretation, readers should refer to the numerical r values shown within each cell.

Correlation analysis showed that telehealth physical therapy willingness, knowledge, and attitude scores were positively associated with the physical function domain and negatively associated with sleep disturbance (Figure 2 and Supplementary Table S4). The strongest positive correlation was observed between knowledge score and physical function domain (r = 0.368), whereas the strongest negative correlation was found between knowledge score and sleep disturbance (r = −0.319). Most other PROMIS-29 domains showed weak or non-significant correlations with telehealth-related variables.

Multivariable linear regression analysis showed that the knowledge score (B = 0.057, β = 0.181, *p* = 0.011) and attitude score (B = 0.063, β = 0.440, *p* < 0.001) were independent predictors of greater willingness to use telehealth physical therapy (Table 3). Based on standardized β values, attitude score demonstrated the strongest relative association with willingness, followed by knowledge score. Barrier score, age, and sex were not significantly associated with willingness (*p* > 0.05). The model explained a moderate proportion of the variance in willingness to use telehealth physical therapy (R^2^ = 0.381; adjusted R^2^ = 0.370; F(5, 303) = 37.25, *p* < 0.001), indicating acceptable explanatory ability for the included predictors.

## 4. Discussion

This study examined the acceptability of telehealth physical therapy among adults with CLBP and explored the factors associated with the willingness to use this form of care. This topic is highly relevant to contemporary rehabilitation practice, where telehealth is increasingly positioned as a means of improving access, reducing logistical barriers, and supporting the non-pharmacological management of musculoskeletal disorders [12]. In the present sample, willingness differed significantly across the provider categories. The highest willingness was observed for dietitian and family doctor services, whereas physical therapy received the lowest willingness score. Knowledge and attitude were positively associated with willingness to use telehealth physical therapy and remained significant in the multivariate model. The regression findings further indicate that attitude had the strongest relative association with willingness to use telehealth physical therapy, followed by knowledge, based on standardized β values. The PROMIS-29 physical function domain was also positively correlated with willingness, whereas barriers, age, and sex were not significant independent predictors. In this study, females had more psychological symptoms and pain interference, while males showed higher social participation. Such sex-related differences in anxiety, depression, fatigue, and pain align with previous research [31]. Despite males facing more telehealth physical therapy barriers, willingness to use telehealth was similar across sexes, suggesting acceptance is influenced more by perceived usefulness and attitudes than by demographics.

The findings indicate that telehealth physical therapy is generally deemed acceptable by adults with CLBP. However, it is approached with greater caution than other forms of remote healthcare, likely because of the traditional perception of physical therapy as a hands-on intervention that requires physical presence. In contrast to counseling, which primarily involves dialogue, physical therapy is frequently associated with function- based, exercise supervision, therapist feedback, and direct physical interactions. Patients may therefore doubt whether telehealth formats can provide the same value as traditional physical therapy. Previous telehealth physical therapy and musculoskeletal telerehabilitation studies have reported that remotely delivered rehabilitation can be feasible and acceptable for selected musculoskeletal conditions, particularly when exercise instruction, therapist feedback, and follow-up are clearly structured. However, these studies have also emphasized that patients may remain cautious when remote care is perceived as unable to replace hands-on assessment, manual guidance, or direct therapist supervision [32,33]. The lower willingness to participate in this study appears to reflect caution rather than a refusal. This interpretation is supported by the barrier findings which have highlighted the continuing concerns about the lack of physical contact and reliability of the equipment, although the privacy concerns have been less pronounced [34,35]. The key finding was that knowledge, and attitudes were independently related to the willingness to use physical therapy in the context of telehealth services. This is clinically relevant as both factors can be modulated. Participants who understood telehealth better and had a more positive attitude towards it were more likely to accept telehealth physical therapy. This suggests that the willingness to use remote care is shaped not only by demographic or clinical factors, but also by the patient’s understanding and interpretation of remote care [36]. From an implementation perspective, these findings suggest that increased patient education, better orientation to telehealth processes, and clearer explanations of what can be achieved with remote physical therapy can increase acceptance. If patients have a more specific understanding of telehealth physical therapy, including its benefits and limitations, they may be more likely to consider it a legitimate part of their care package [37].

A positive correlation between the PROMIS-29 physical function domain and willingness to use telehealth physical therapy. Participants with better physical function domain were more willing to consider telehealth, perhaps because they felt more able to do exercises, follow therapist instructions and manage their own health without the physical help of a therapist. However, alternative explanations should be considered. Participants with better physical function may have greater confidence in performing home-based exercises independently, a lower perceived need for hands-on assessment, fewer safety concerns, or greater familiarity with digital platforms. Conversely, individuals with poorer function may prefer in-person care because of concerns regarding supervision, movement correction, pain aggravation, or the need for direct therapist support. Therefore, this association should be interpreted cautiously and not as evidence that physical function directly determines telehealth willingness. On the other hand, an individual with reduced functional capacity may perceive a greater need for hands-on examinations, close supervision or therapist support, which makes remote rehabilitation less appropriate [38]. This finding has practical implications for clinical decision-making process. Telehealth physical therapy may be most suitable for selected patients who can safely participate in guided home-based treatment, while patients with more functional limitations may benefit more from a hybrid approach or an initial in-person evaluation prior to remote monitoring. A comparison between different types of providers also provides useful insights. The greater willingness to use telehealth for the services of dietitians and family doctors than for physical therapy suggests that acceptance is shaped in part by the perception of the various professions. Consultation-based services can be easier to imagine for patients in virtual environments. In contrast Physical therapy is closely associated with physical examinations and direct therapeutic contact in the public mind. These occupational perceptions suggest that the introduction of telehealth in physical therapy should not be a simple replication of strategies applied in other health care areas. Instead, patient preparation and anticipation may be particularly important in physical therapy, where the benefits of remote care may be less apparent than in other fields. Sex differences should be interpreted cautiously. Females represented 90.0% of the sample, whereas males represented only 10.0%, resulting in a marked sex imbalance. Therefore, the observed sex-based differences in the PROMIS-29 domains, knowledge, perceived barriers, and telehealth willingness may be less stable and should not be interpreted as definitive evidence of sex-related differences. The small number of male participants may have limited the statistical power and precision of these comparisons, and future studies with more balanced sex distribution are needed to confirm these findings. Females reported slightly higher knowledge scores, while males reported higher barrier scores. However, in the modified model sex was not an independent predictor of willingness and no significant sex difference in willingness to use telehealth physical therapy was observed. This suggests that telehealth perceptions are more influential than sex in shaping attitudes towards telehealth practice. This supports the priority given to modifiable perceptual factors such as knowledge and attitudes rather than relying heavily on demographic profiling in the design of telehealth services [39].

Uncertainty about the actual nature of this type of care may contribute to the a lack of willingness to use physical therapy in the context of telehealth. From a quick video consultation to a structured physical-therapy program, the participants could envision entirely different services. Because of the online nature of the survey, the sample may have included more individuals who were more used to digital devices than the low-pain population, which may have resulted in a more positive view of telehealth. The sample was predominantly female (90.0%) and relatively young, with a mean age of 26.06 ± 9.29 years. This demographic profile may not fully reflect the broader CLBP population, which includes a wider age range and a more balanced sex distribution. Therefore, the reported telehealth acceptability scores may have been influenced by greater digital literacy, voluntary online participation, health-related engagement, and mild pain intensity in the recruited participants. These factors may have led to overestimation of telehealth willingness and should be considered when interpreting the findings. Therefore, rather than being representative of the wider adult population with CLBP, these results should be interpreted as reflecting the characteristics of the recruited study sample. Nevertheless, a strength of the study is that participants had diagnosis of CLBP, defined using pain duration, pain frequency, and symptom chronicity, which helped reduce heterogeneity of the study population. Although the participants met the study definition of CLBP, the relatively young age of the sample, generally low pain intensity, and variation in symptom frequency and duration may not fully reflect the broader clinical population typically affected by persistent or disabling CLBP. These characteristics may have influenced the participants perceived need for in-person physical therapy, confidence in self-management, and willingness to consider telehealth-based care. Therefore, the reported acceptability scores should be interpreted cautiously and should not be generalized to older adults, individuals with higher pain severity, or patients with more persistent and functionally limiting CLBP. These findings may inform future telehealth physical therapy implementation planning; however, they should be interpreted cautiously because the willingness to use telehealth does not necessarily translate into actual utilization, adherence, satisfaction, or clinical benefit. Patients with limited knowledge or less positive attitudes may benefit from brief education, platform orientation, and clear explanation of remote care. Screening for barriers, functional limitations, and home-exercise confidence may help identify patients who need closer supervision, additional support, or a hybrid model with an initial in-person assessment and remote follow up. This interpretation is consistent with the previous telehealth physical therapy literature suggesting that remote rehabilitation may be most appropriate when patients receive adequate education, technical orientation, exercise monitoring, and the option of hybrid care when in-person assessment is clinically required [33,40].

There are several limitations to be taken into account. First, the sample size achieved (n = 309) was lower than the a priori target of 384 participants. This shortfall may have reduced the precision of the prevalence and descriptive estimates and increased uncertainty around subgroup comparisons. This study assessed stated willingness to use telehealth physical therapy rather than actual utilization, adherence, satisfaction, clinical effectiveness, or implementation success. Therefore, the findings should be interpreted primarily as analytical associations within the recruited sample rather than as population-level estimates for all adults with CLBP. Second, recruitment relied on a self-selected online sample, which increased the risk of selection bias. Participants with greater digital access, higher confidence in using online platforms, or more favorable views toward telehealth may have been more likely to participate; therefore, acceptability estimates may be higher than those expected in less digitally engaged CLBP populations. Third, the findings should not be generalized to the wider adult population with low back pain because the sample was predominantly female and relatively young. In particular, females represented 90.0% of the sample, leaving a comparatively small number of male participants. This marked sex imbalance may have influenced the estimates of telehealth acceptability, perceived barriers, and sex-based comparisons in this study. Because male participants represented only 10.0% of the sample, sex-based comparisons may have been underpowered and should be interpreted cautiously. The predominance of female respondents may also have affected the overall pattern of PROMIS-29 psychological symptoms, pain interference, perceived barriers, and willingness estimates, limiting the extent to which these findings can be generalized to male or more sex-balanced CLBP populations. Fourth, the decision should be interpreted with caution, as the variables of knowledge, attitudes and barriers, although derived from items on the Likert scale, were analyzed as composite scores across multiple items and treated as roughly continuous. Moreover, the observed associations should not be interpreted as predictive effects as the cross-sectional design of the study prevents a causal relationship. Therefore, the association between knowledge, attitudes, barriers, PROMIS-29 domains, and willingness to use telehealth physical therapy should be interpreted as associations only, and no causal or directional conclusions can be drawn from this study. The results may also be affected by self-report bias, response bias, and social desirability biases, as all measures were self-reported. Although the Arabic questionnaire wording was reviewed for clarity and conceptual equivalence, the Arabic TMPQ and PROMIS-29 versions were not formally revalidated in the present sample of patients with CLBP, which should be considered when interpreting questionnaire-based findings. Finally, willingness to use telehealth physical therapy was measured as a stated preference rather than actual utilization, adherence, satisfaction, or clinical effectiveness. Therefore, the results should be interpreted with caution as findings from a specific recruited sample rather than as population-level estimates.

Future research should include randomized trials comparing in-person, hybrid, and fully remote models of treatment for low back pain; which would be of enormous value. It would also be useful to identify whether training interventions can improve telehealth preparedness by increasing awareness and encouraging a more positive attitude prior to treatment. Qualitative research can also help to explain why some patients remain reluctant, especially regarding limited physical contact, confidence in remote evaluation, and trust in digital tools. This evidence would support a more refined and patient-oriented implementation of telehealth physical therapy in musculoskeletal care.

## 5. Conclusions

The study found that participants with CLBP who had better knowledge of telehealth and more positive attitudes toward it showed greater willingness to use telehealth physical therapy. Clinically, these findings suggest that brief patient education, pre-session orientation to telehealth platforms, screening for perceived barriers, and clear explanation of remote exercise supervision may support readiness for telehealth-based physical therapy. However, this study assessed stated willingness and acceptability, not actual telehealth utilization, adherence, satisfaction, or treatment effectiveness. Causal effects on actual uptake cannot be inferred from this cross-sectional study. These findings should also be interpreted cautiously because voluntary online recruitment and the predominantly female, relatively young sample may limit generalizability to the broader CLBP population. Future longitudinal and interventional studies should examine whether education-based strategies improve actual telehealth use, adherence, satisfaction, and clinical outcomes in more diverse populations with CLBP.

## Figures and Tables

**Table 1:** Demographic and clinical characteristics of the participants (N = 309).

Characteristic	Category	Value
Age (years)	Mean ± SD	26.06 ± 9.29
Sex	Female	277 (90.0%)
	Male	32 (10.0%)
Body mass index (kg/m^2^)	Mean ± SD	24.12 ± 5.82
Marital status	Single	235 (76.0%)
	Married	67 (22.0%)
	Other	7 (2.0%)
Smoking status	No	294 (95.1%)
	Yes	15 (4.9%)
Education level	Bachelor	197 (63.8%)
	High school	55 (17.8%)
	Diploma	24 (7.8%)
	Master	17 (5.5%)
	PhD	12 (3.9%)
	Other	4 (1.3%)
Chronic diseases	No	256 (82.8%)
	Yes	53 (17.2%)
History of other musculoskeletal disorders	No	178 (57.6%)
	Yes	131 (42.4%)
Currently experiencing low back pain	Yes	309 (100.0%)
Current level of low back pain (0–10 NPRS)	Mean ± SD	2.78 ± 2.03
Lowest pain in last 24 h	Mean ± SD	1.56 ± 1.99
Worst pain in last 24 h	Mean ± SD	2.78 ± 2.85
Time since first episode of low back pain	1 year or less	182 (58.9%)
	2–5 years	63 (20.4%)
	5 years or more	64 (20.7%)
Duration of persistent low back pain problem	3 months	97 (31.4%)
	3–6 months	34 (11.0%)
	6 months to 1 year	54 (17.5%)
	1–5 years	45 (14.6%)
	>5 years	45 (14.6%)
	None/Other	34 (11.0%)
Frequency of low back pain in last 6 months	Less than half days	152 (49.4%)
	At least half days	73 (23.7%)
	Daily	63 (20.5%)
	No	20 (6.5%)

Note: History of other musculoskeletal disorders refers to musculoskeletal conditions other than the current low back pain complaint. Abbreviations: SD, standard deviation; NPRS, numeric pain rating scale.

**Table 2:** Comparison of patient willingness across healthcare providers.

Provider Type	Mean Willingness (1–5)	Standard Deviation
Dietitian	3.86	1.15
Family Doctor	3.84	1.11
Mental Health Therapist	3.66	1.17
Urgent Care	3.54	1.16
Physical Therapy	3.52	1.13
Friedman Test (*p*-value)	<0.001	

**Table 3:** Multivariable linear regression of variables independently associated with telehealth physical therapy willingness.

Predictor	B	Standardized β	95% CI	*p*-Value
Knowledge score	0.057	0.181	0.013 to 0.102	0.011
Attitude score	0.063	0.440	0.044 to 0.082	<0.001
Barrier score	−0.028	−0.087	−0.057 to 0.001	0.061
Age (years)	0.000	0.000	−0.001 to 0.001	0.594
Sex (Male vs. Female)	−0.054	−0.015	−0.389 to 0.281	0.752

Model fit: R^2^ = 0.381; adjusted R^2^ = 0.370; F(5, 303) = 37.25, *p* < 0.001. The model explained 38.1% of the variance in willingness to use telehealth physical therapy. B = unstandardized regression coefficient; β = standardized regression coefficient; CI = confidence interval for B; reference category for sex = female. Positive coefficients indicate greater willingness to use telehealth physical therapy.

## Data Availability

The datasets analyzed in the current study are available from the corresponding author on reasonable request.

## Supplementary Materials

The following supporting information can be downloaded at: https://www.mdpi.com/article/10.3390/healthcare14142036/s1, Table S1: Frequency Distribution of Items in the Telemedicine Perception Questionnaire (TMPO) (N = 309). Table S2: Bivariate Comparison of Domain Scores by Sex. Table S3: PROMIS-29 v2.0 T-scores by sex. Table S4: Correlation matrix of PT willingness and TMPQ scores with PROMIS-29 v2.0 domains.

## Abbreviations

The following abbreviations are used in this manuscript:

CLBP	Chronic Low Back Pain
NPRS	Numeric Pain Rating Scale
PROMIS-29	Patient-Reported Outcomes Measurement Information System
QoL	Quality of Life
TMPQ	Telemedicine Perception Questionnaire

## References

[1] Meshram S.C., Verma C. (2025). Comparative analysis of attitudes and threat perceptions towards physical activities in individuals with and without low back pain. Bull. Fac. Phys. Ther..

[2] El-Tallawy S.N., Nalamasu R., Salem G.I., LeQuang J.A., Pergolizzi J.V., Christo P.J. (2021). Management of Musculoskeletal Pain: An Update with Emphasis on Chronic Musculoskeletal Pain. Pain Ther..

[3] Dowell D., Ragan K., Jones C.M., Baldwin G., Chou R. (2022). CDC Clinical Practice Guideline for Prescribing Opioids for Pain—United States, 2022. MMWR Recomm. Rep..

[4] Mobasheri A. (2022). Self-Healing: A Novel and Integrated Multimodal Concept for the Management of Musculoskeletal Pain. J. Pain Res..

[5] Bareiss S.K., Nare L., McBee K. (2019). Evaluation of pain knowledge and attitudes and beliefs from a pre-licensure physical therapy curriculum and a stand-alone pain elective. BMC Med. Educ..

[6] Tatta J., Spoto M.M., Lorenzetti J. (2022). Pain Education Training in New York State Physical Therapy Programs: What We Do Well, Where the Gaps Are, and What Can Be Improved. Internet J. Allied Heal. Sci. Pract..

[7] Shayo M.J., Shayo P., Haukila K.F., Norman K., Burke C., Ngowi K., Goode A.P., Allen K.D., Wonanji V.T., Mmbaga B.T. (2023). Expanding access to rehabilitation using mobile health to address musculoskeletal pain and disability. Front. Rehabil. Sci..

[8] Fritz J.M., Ford I., George S.Z., Vinci de Vanegas L., Cope T., Burke C.A., Goode A.P. (2024). Telehealth delivery of physical therapist-led interventions for persons with chronic low back pain in underserved communities: Lessons from pragmatic clinical trials. Front. Pain Res..

[9] Akyürek G., Bektaş S.A. (2023). Telerehabilitation: An Updated View of Practices, Cost Analysis, and Client Perceptions. Internet J. Allied Heal. Sci. Pract..

[10] English C., Fritz N.E., Gomes-Osman J. (2023). Telehealth Models of Service Delivery—A Brave New World. J. Neurol. Phys. Ther..

[11] Johnson M.A., Khilnani T., Hyun A., Amen T.B., Varady N.H., Nwachukwu B.U., Dines J.S. (2025). The State of Telemedicine, Telerehabilitation, and Virtual Care in Musculoskeletal Health: A Narrative Review. HSS J..

[12] Smith A.D.M., Innes S. (2025). Patient and clinician perceptions of telehealth in musculoskeletal physiotherapy services—A systematic review of the evidence-base. PLoS Digit. Health.

[13] Moecke D.P., Holyk T., Maddocks S., Campbell K.L., Ho K., Camp P.G. (2024). Physical Therapists’ Perspectives on the Use of Telehealth With First Nations Peoples in Canada: A Qualitative Study. Phys. Ther..

[14] Rossetto F., Borgnis F., Isernia S., Foglia E., Garagiola E., Realdon O., Baglio F. (2023). System Integrated Digital Empowering and teleRehabilitation to promote patient Activation and well-Being in chronic disabilities: A usability and acceptability study. Front. Public Health.

[15] Sahan F., Gutermuth A., Müller J.A., Muth T., Panitz K., Apolinário-Hagen J. (2026). Acceptance and Use of Digital Health Technologies among Physiotherapists in Germany: A Web-Based Cross-Sectional Survey. BMC Health Serv. Res..

[16] Vandenbroucke J.P., von Elm E., Altman D.G., Gøtzsche P.C., Mulrow C.D., Pocock S., Poole C., Schlesselman J.J., Egger M., STROBE Initiative (2014). Strengthening the Reporting of Observational Studies in Epidemiology (STROBE): Explanation and elaboration. Int. J. Surg..

[17] Arévalo M., Brownstein N.C., Whiting J., Meade C.D., Gwede C.K., Vadaparampil S.T., Tillery K.J., Islam J.Y., Giuliano A.R., Christy S.M. (2022). Strategies and Lessons Learned During Cleaning of Data from Research Panel Participants: Cross-sectional Web-Based Health Behavior Survey Study. JMIR Form. Res..

[18] Tufan U.E., Aktürk Z. (2021). The Strengthening the Reporting of Observational Studies in Epidemiology (Strobe) Statement: Guidelines for Reporting Observational Studies. Alatoo Acad. Stud..

[19] Carter S.M., Shih P., Williams J., Degeling C., Mooney-Somers J. (2021). Conducting Qualitative Research Online: Challenges and Solutions. Patient.

[20] Stratton S.J. (2021). Population Research: Convenience Sampling Strategies. Prehosp. Disaster Med..

[21] Green S.B. (1991). How Many Subjects Does It Take to Do a Regression Analysis. Multivar. Behav. Res..

[22] Krejcie R.V., Morgan D.W. (1970). Determining sample size for research activities. Educ. Psychol. Meas..

[23] Deyo R.A., Jarvik J.G., Chou R. (2014). Low back pain in primary care. BMJ.

[24] Childs J.D., Piva S.R., Fritz J.M. (2005). Responsiveness of the numeric pain rating scale in patients with low back pain. Spine.

[25] Su X.V., Isnani S., Khadijah W.M.S.S., Shareezan H.S., Lau L.K., Maziah I., Tajuddin N.A.A., Chan C.W. (2024). Perception of primary care doctors towards telemedicine in Kuching, Sarawak: A cross-sectional study. Malays. Fam. Physician.

[26] Harst L., Lantzsch H., Scheibe M. (2019). Theories predicting end-user acceptance of telemedicine use: Systematic review. J. Med. Internet Res..

[27] Altmann P., Ivkic D., Ponleitner M., Leutmezer F., Willinger U., Schmoeger M., Berger T., Bsteh G., Löffler-Stastka H. (2022). Individual Perception of Telehealth: Validation of a German Translation of the Telemedicine Perception Questionnaire and a Derived Short Version. Int. J. Environ. Res. Public Health.

[28] Hays R.D., Herman P.M., Rodriguez A., Edelen M.O. (2023). Comparison of patient-reported outcomes measurement information system (PROMIS^®^)-29 and PROMIS global physical and mental health scores. Qual. Life Res..

[29] Hwang M.C., Bell C., Farran Y., Ogdie A., Green C., Reveille J. (2024). Reliability and validity of the PROMIS-29 health profile in Ankylosing Spondylitis patients: A cross-sectional study. Medicine.

[30] Wild D., Grove A., Martin M., Eremenco S., McElroy S., Verjee-Lorenz A., Erikson P. (2005). Principles of good practice for the translation and cultural adaptation process for patient-reported outcomes measures: Report of the ISPOR Task Force for Translation and Cultural Adaptation. Value Health.

[31] Tlach L., Hampel P. (2010). Geschlechtsunterschiede in psychosozialen Kennwerten bei Patienten in der stationären orthopädischen Rehabilitation von chronisch unspezifischen Rückenschmerzen [Gender differences in psychosocial variables in inpatient orthopedic rehabilitation of chronic low back pain]. Psychother. Psychosom. Med. Psychol..

[32] Dario A.B., Moreti Cabral A., Almeida L., Ferreira M.L., Refshauge K., Simic M., Pappas E., Ferreira P.H. (2017). Effectiveness of telehealth-based interventions in the management of non-specific low back pain: A systematic review with meta-analysis. Spine J..

[33] Cottrell M.A., Galea O.A., O’Leary S.P., Hill A.J., Russell T.G. (2017). Real-time telerehabilitation for the treatment of musculoskeletal conditions is effective and comparable to standard practice: A systematic review and meta-analysis. Clin. Rehabil..

[34] Patel M., Berlin H., Rajkumar A., Krein S.L., Miller R., DeVito J., Roy J., Punch M., Ellimootti C., Peahl A.F. (2023). Barriers to Telemedicine Use: Qualitative Analysis of Provider Perspectives During the COVID-19 Pandemic. JMIR Hum. Factors.

[35] Grace S., Engel R., Mastronardo C., Muddle L., Fleischmann M., Vaughan B., Fazalbhoy A. (2023). Perceptions of Australian osteopaths on the use of telehealth for patient care: Barriers and enablers for implementation. Int. J. Osteopath. Med..

[36] Javed A., Yamin G. (2024). Patient Perspectives on the Need for Telehealth Services in Rehabilitation. J. Health Rehabil. Res..

[37] McLaughlin K.H., Minick K.I., Fritz J.M., Tannahill N., Spar A., Feinsilver M., Weber M., Opoku E., Adams J., Skolasky R.L. (2026). Physical Therapists’ Perceptions of Telerehabilitation for Patients with Musculoskeletal Conditions in the Post-Pandemic World. Telemed. J. e-Health.

[38] Jaswal S., Lo J., Sithamparanathan G., Nowrouzi-Kia B. (2023). The era of technology in healthcare: An evaluation of telerehabilitation on patient outcomes—A systematic review and meta-analysis protocol. Syst. Rev..

[39] Ghorbal A.B., Elbatal I., Aldukeel A.R., Elshabrawy A., Ibrahim S.A., El-Zayat N.I.A., Zakaria S., El-Wahab H.A.A., Sabry D.M., Elsayed T. (2025). Perceived ease of use of telehealth services and associated factors in Saudi Arabia: A cross-sectional study. PLoS ONE.

[40] Lee A.C., Davenport T.E., Randall K. (2018). Telehealth Physical Therapy in Musculoskeletal Practice. J. Orthop. Sports Phys. Ther..

