# Supplementary material for: Acceptability of Telehealth Physical Therapy in Adults with Chronic Low Back Pain: A Multivariable Analysis of Knowledge, Attitudes, Barriers, and Biopsychosocial Health Factors
Source: Healthcare (Basel). 2026 Jul 8;14(14):2036. doi: 10.3390/healthcare14142036 (PMC13409947; doi:10.3390/healthcare14142036)
Supplement: Supplementary file 1 [file healthcare-14-02036-s001.zip › healthcare-4378546-supplementary.pdf]

Table (S1): Frequency Distribution of Items in the Telemedicine Perception Questionnaire (TMPO) (N = 309)

Domain / Item	Strongly Disagree	Disagree	Don't Know	Agree	Strongly Agree
**Knowledge Items**					
Can be used for diagnosis?	14.2%	11.0%	12.3%	44.0%	18.4%
Can be used for assessment?	23.0%	11.0%	14.9%	32.4%	18.8%
**Attitude Items**					
Saves my time	1.9%	5.5%	8.1%	52.1%	32.4%
Saves my money	1.9%	7.8%	14.6%	49.2%	26.5%
Suitability as healthcare	4.2%	11.0%	14.9%	51.8%	18.1%
**Barrier Items**					
May violate my privacy	33.0%	39.2%	12.9%	11.7%	3.2%
Lack of physical contact	7.4%	27.8%	16.8%	32.7%	15.2%
Cannot trust equipment	12.0%	30.1%	18.8%	31.7%	7.4%

Table (S2): Bivariate Comparison of Domain Scores by Sex

Outcome	Female (n = 277)	Male (n = 32)	p-value
**Knowledge Score**	14.3 ± 3.6	13.2 ± 3.3	0.049
**Attitude Score**	42.0 ± 8.0	41.0 ± 7.0	0.700
**Barriers Score**	14.7 ± 3.6	16.1 ± 2.5	0.043
**PT Willingness**	3.53 ± 1.13	3.38 ± 1.16	0.452

Table (S3): PROMIS-29 v2.0 T-scores by sex

PROMIS-29	All participants	Females	Males	p-value
Physical function	46.50 ± 8.32	46.59 ± 8.39	45.76 ± 7.79	0.573
Anxiety	61.02 ± 8.59	61.70 ± 8.40	55.16 ± 8.14	<0.001
Depression	57.10 ± 9.62	57.73 ± 9.51	51.61 ± 8.94	<0.001
Fatigue	56.93 ± 9.20	57.64 ± 8.81	50.80 ± 10.29	<0.001
Sleep disturbance	50.19 ± 8.43	50.08 ± 8.70	51.14 ± 5.61	0.347
Ability to participate in social roles	47.69 ± 8.40	47.26 ± 8.16	51.36 ± 9.59	0.026
Pain interference	58.78 ± 6.52	59.17 ± 6.27	55.38 ± 7.68	0.011
Pain intensity (0–10)	2.57 ± 2.45	2.59 ± 2.49	2.44 ± 2.09	0.707

Table (S4): Correlation matrix of PT willingness and TMPQ scores with PROMIS-29 v2.0 domains

**Primary score**	**Physical function**	**Anxiety**	**Depression**	**Fatigue**	**Sleep disturbance**	**Ability to participate in social roles**	**Pain interference**	**Pain intensity, 0–10 NRS**
PT willingness score	0.319 (<0.001)	-0.038 (0.509)	-0.079 (0.164)	-0.058 (0.311)	-0.306 (<0.001)	-0.003 (0.967)	0.116 (0.089)	-0.043 (0.452)
Knowledge score	0.368 (<0.001)	-0.036 (0.532)	-0.105 (0.066)	-0.043 (0.454)	-0.319 (<0.001)	0.045 (0.491)	0.099 (0.144)	-0.073 (0.199)
Attitude score	0.350 (<0.001)	-0.036 (0.526)	-0.090 (0.114)	-0.034 (0.547)	-0.274 (<0.001)	0.039 (0.556)	0.119 (0.079)	-0.060 (0.291)
Barriers score	0.037 (0.521)	-0.016 (0.774)	-0.038 (0.511)	0.048 (0.396)	0.045 (0.433)	-0.067 (0.304)	-0.018 (0.793)	-0.010 (0.858)

* p<0.05, ** p<0.01, *** p<0.001.
